# The impact of stigma on the provision and coordination of dementia care

**DOI:** 10.1002/alz.083423

**Published:** 2025-01-09

**Authors:** Roxanne Jacobs

**Affiliations:** ^1^ University of Cape Town, Cape Town, Western Cape South Africa

## Abstract

**Background:**

Care and support for people with dementia and their families in South Africa are largely inadequate. Responses to dementia are driven by a widespread lack of understanding of dementia amongst the general public, communities, and within local health, policy‐ and social care systems. This presentation will focus on the findings of a situational analysis completed within the STRIDE project (i.e. strengthening responses to dementia in developing countries) that provided an evidence base to inform priority‐setting for strengthening responses to dementia in South Africa.

**Method:**

Evidence was generated on current provisions for older persons and their families through: (1) an in‐depth desk review; (2) multi‐stakeholder interviews; and (3) a SWOT‐analysis (strengths, weaknesses, opportunities, and threats).

**Result:**

Our findings highlight the gaps in current service provision from diagnosis through to follow up support and end‐of‐life care. The findings show how structural factors (including stigma and discrimination) create barriers to diagnosis, support and care.

**Conclusion:**

There is an urgent need for inter‐sectoral policy development in South Africa to support adequate health, social care, and long‐term care solutions for people with dementia and their families. This situational analysis provides an evidence base to inform priority‐setting and strengthening responses to dementia in South Africa.

